# Genotypic and functional properties of early infant HIV-1 envelopes

**DOI:** 10.1186/1742-4690-8-67

**Published:** 2011-08-15

**Authors:** Michael Kishko, Mohan Somasundaran, Frank Brewster, John L Sullivan, Paul R Clapham, Katherine Luzuriaga

**Affiliations:** 1 Graduate School of Biomedical Sciences, University of Massachusetts Medical School, Worcester, MA, USA; 2 Department of Pediatrics, University of Massachusetts Medical School, Worcester, MA, USA; 3 Program in Molecular Medicine, University of Massachusetts Medical School, Worcester, MA, USA

## Abstract

**Background:**

Understanding the properties of HIV-1 variants that are transmitted from women to their infants is crucial to improving strategies to prevent transmission. In this study, 162 full-length *envelope *(*env*) clones were generated from plasma RNA obtained from 5 HIV-1 Clade B infected mother-infant pairs. Following extensive genotypic and phylogenetic analyses, 35 representative clones were selected for functional studies.

**Results:**

Infant quasispecies were highly homogeneous and generally represented minor maternal variants, consistent with transmission across a selective bottleneck. Infant clones did not differ from the maternal in *env *length, or glycosylation. All infant variants utilized the CCR5 co-receptor, but were not macrophage tropic. Relatively high levels (IC_50 _≥ 100 μg/ml) of autologous maternal plasma IgG were required to neutralize maternal and infant viruses; however, all infant viruses were neutralized by pooled sera from HIV-1 infected individuals, implying that they were not inherently neutralization-resistant. All infant viruses were sensitive to the HIV-1 entry inhibitors Enfuvirtide and soluble CD4; none were resistant to Maraviroc. Sensitivity to human monoclonal antibodies 4E10, 2F5, b12 and 2G12 varied.

**Conclusions:**

This study provides extensive characterization of the genotypic and functional properties of HIV-1 *env *shortly after transmission. We present the first detailed comparisons of the macrophage tropism of infant and maternal *env *variants and their sensitivity to Maraviroc, the only CCR5 antagonist approved for therapeutic use. These findings may have implications for improving approaches to prevent mother-to-child HIV-1 transmission.

## Background

Mother-to-child HIV-1 transmission is the primary mode of pediatric infection. Over 50% of HIV-1 infected individuals around the world are women in their childbearing years [1,2]. In the absence of intervention, more than a third of the children born to infected mothers acquire HIV-1 through mother-to-child transmission (MTCT) [3-5]. This accounts for up to 14% of all HIV-1 transmission [1,5], with 370,000 infants infected in 2009. MTCT can occur during gestation, at delivery and through breastfeeding. Seventy-five percent of HIV-1 infected children die by the age of 3 years, accounting for up to 20% of all HIV-1 related deaths [6,7]; in resource-limited settings, HIV-1 accounts for one third of all deaths among children under five [1].

Studies in multiple cohorts, across several clades, have demonstrated that a marked restriction in the diversity of founder viruses in blood and plasma is a hallmark of mucosal HIV-1 infection, including sexual transmission [8-12] and MTCT [13]. This restricted diversity suggests either the transmission or post-transmission amplification of a single donor variant in the majority of recipients [3,14-16]. The genetic and biologic determinants of the transmission bottleneck are largely unknown.

The *env *glycoprotein (gp160*) *engages the HIV-1 receptor and co-receptors, mediating virus entry into cells [17], and is the primary target for neutralizing antibodies. *Env *is also the most variable HIV-1 gene. We therefore set out to extensively characterize the genotypes and phenotypes of full-length *env *molecular clones from HIV-1 infected mother-infant pairs. Better understanding of the genotypic and functional properties of transmitted *env *variants may facilitate the development of improved strategies to prevent MTCT.

## Results

### Phylogeny of envelope sequences

**Figure 1:**
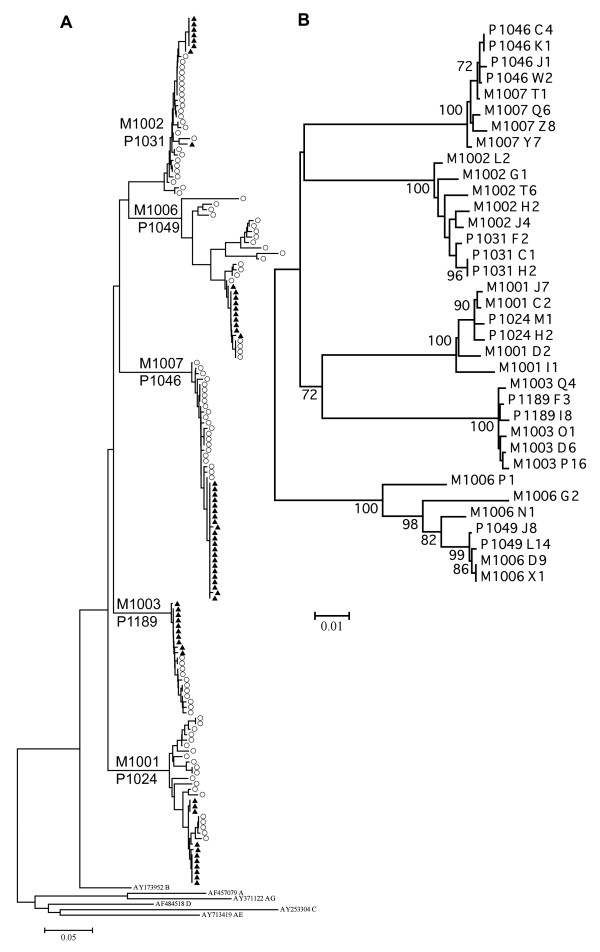
**Evolutionary relationships of HIV-1 *env *clones**. Evolutionary history was inferred using the Neighbor-Joining method. **(A) **V1-V5 nucleotide sequences of cloned *env *and subtype reference sequences. Filled triangle = infant, empty circle = maternal sequence. **(B) **Full length gp160 nucleotide sequences. M = maternal, P = infant. The percentage of replicate trees in which the associated sequences clustered together >70% of the time in the bootstrap test (1000 replicates) are shown to the left of branches in **(B)**. The evolutionary distances were computed using the Kimura 2-parameter method. All positions containing gaps and missing data were eliminated from the dataset. Horizontal scale bars represent **(A) **5%, or **(B) **1% genetic distance.

Full-length *env *genes were amplified from mother and infant patient plasma HIV-1 RNA (Table 1). At least 10 clones were generated for each subject; 88% of *env *clones proved functional, with no significant differences in functionality between mothers and infants detected within or across transmission pairs (data not shown). A total of 162 functional maternal and infant *env *clones, each from an independent limiting dilution RT-PCR, were obtained and sequenced through the V1-V5 regions of the envelopes. A neighbor-joining tree was constructed by alignment of these nucleotide sequences (Figure 1A). For one patient (P1031), three clones were sequenced through V1-V3 only and are not included in the tree. The resulting tree revealed clear epidemiological linkage within each mother-infant pair, with no evidence of cross-pair or other contamination. Maximum likelihood trees and Highlighter alignments of non-gap stripped sequences were used to confirm phylogeny and select representative clones (data not shown).

**Table 1:** Clinical and laboratory status of study participants

Subject*^a^*	Birth year	Sample timing	Plasma viral load (copies/ml)	CD4	CD8	CD4:CD8	No. of *env *clones	No. of pseudo viruses	ART status
M1003		0	14158	466	932	0.50	12	4	None
P1189	1994	31	311538	2872	1975	1.45	10	2	None
M1002		28	ND	872	1225	0.71	25	5	None
P1031	1992	54	685169	2147	927	2.32	11	3	None
M1001		2	26000	534	726	0.74	19	4	None
P1024	1990	51	750000	3312	4504	0.74	11	2	None
M1007		-8	ND	870	1176	0.74	22	4	ZDV
P1046	1995	66	1229730	2573	1693	1.52	22	4	ZDV*
M1006		-33	260541	134	403	0.33	20	5	ZDV
P1049	1999	30	647919	ND	ND	ND	10	2	ZDV*

*^a^*M, Mother; P, Infant. ZDV, Zidovudine administered to mother or infant to prevent MTCT. ND, Not determined. Timing of samples used for cloning in days after delivery; negative numbers indicate days before delivery.

At least 2 clones were selected from each infant: the closest to and farthest from the consensus of the subject. In two cases where the infants were clearly infected with two maternal variants (P1031 and P1024; Figure 1A), clones from the major infant variant were selected as above, and the clone closest to the consensus of the minor infant variant was also included. At least four maternal clones were selected from each subject to sample the breadth of their quasispecies. Using Maximum Likelihood Trees, a maternal clone was selected from each of the two branches closest to the infant, and two additional clones were chosen from distantly related branches (data not shown). Full-length gp160 sequences of both DNA strands were obtained for the selected clones.

Full-length *env *sequences were obtained for all selected clones (Figure 1B), and the consensus gp160 sequence was determined for each infant. Of the 13 infant clones selected, four were identical to their infant's gp160 consensus. Eight clones differed from the consensus by two amino acids or less, one differed by three, and one (P1024 H2) differed by six. For two randomly selected infants (P1189 and P1049), consensus gp160 sequences generated by SGA were identical to those obtained by endpoint dilution PCR (data not shown). Phylogenetic analyses confirmed that all subjects were infected with subtype B.

**Figure 2:**
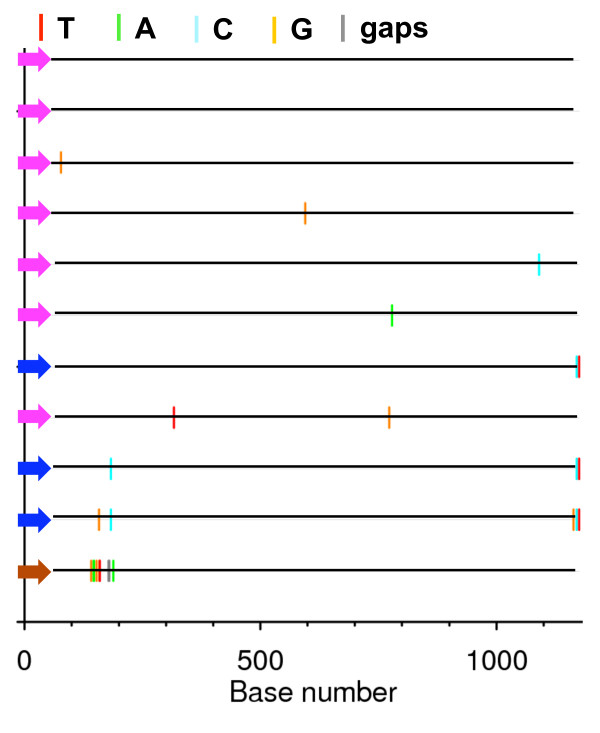
**Highlighter analysis of infant P1024 V1-V5 sequences**. The subject quasispecies consists of three variants. Sequences belonging to the same variant are indicated by colored arrows. Pink and blue variants arose from transmission of two very closely related maternal viruses, or by post-transmission diversification. The brown variant arose from transmission of a distinct maternal virus.

**Figure 3:**
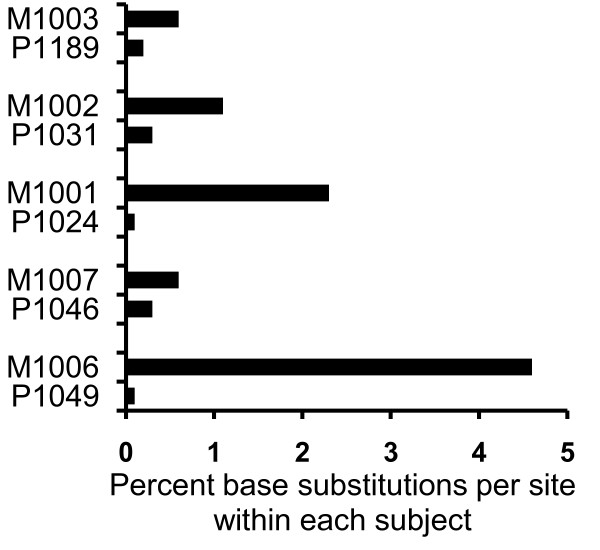
**Infant quasispecies are more homogeneous than maternal**. The percent of base substitutions per site over the V1-V5 region for each subject were computed using the Kimura 2-parameter method in the MEGA4 software program.

Visual inspection of phylogenetic trees (Figure 1 and 2) and Highlighter alignments (data not shown) of each mother-infant pair demonstrated probable transmission of a single maternal variant to infants P1189, P1049, and P1046, two variants to infant P1031 and two or three to infant P1024. Of the variants transmitted to P1024, two arose from very closely related viruses, or through post-transmission diversification (Figure 2). The relationship between maternal and infant quasispecies was further analyzed based on the paradigm described by Haaland *et al*. [18]. The number of amino acids differing between each infant variant and the most closely related maternal sequence in the V1-V5 region were determined, as were the number of maternal sequences differing from an infant variant by less than three amino acids (Table 2). A maternal sequence differing from an infant variant by less than three amino acids likely gave rise to that variant. If such sequences represent less than 5% of the maternal quasispecies, a minor maternal variant was likely transmitted to the infant [18]. Infant P1024 was apparently infected with two or three minor variants of the maternal quasispecies, infant P1049 with a single major variant, infant P1031 with two minor variants, while infants P1189 and P1046 each received a single minor variant (Table 2). Infant sequences were more homogeneous than maternal, with the mean diversity, measured by number of base substitutions per site within each subject ranging from 0.1 to 0.3% among infants, and 0.6 to 4.6% among mothers (Figure 3).

**Table 2:** Relationship of maternal and infant V1-V5 sequences

Infant	Sequences analyzed*^a^*	Differences*^b^*	Less than 3 differences*^c^*
P1189	12	1	1
P1031	25	3	0
		11	0
P1024	19	5	0
		3	0
		3	0
P1046	22	1	1
P1049	20	2	4

*^a^*Number of maternal sequences analyzed.

*^b^*Number of amino acids that differ between an infant variant consensus sequence and the most closely related maternal sequence.

*^c^*Number of maternal sequences differing from the infant variant consensus by less than 3 amino acids.

The consensus sequence of clones amplified shortly following transmission from a subject infected with a single donor variant represents the sequence of the transmitted/founder virus [18]. We compared maternal gp160 sequences to the consensus of each infant variant to determine how closely clones selected for their similarity to infant *env *approached the transmitted/founder sequence. Maternal clones most closely related to their infants were; M1003 P16 which differed from the infant consensus by three amino acid substitutions, M1001 J7 which differed by four amino acids substitutions, M1007 T1 which differed by three amino acids, M1006 X1 which differed by three substitutions, and M1002 J4 which differed by 15 amino acids. No maternal sequence was identical to the consensus of an infant variant. We then compared the maternal sequences to each individual sequence amplified from her infant and did not detect any maternal sequence identical to any infant sequence.

### *Env *V1-V5 length, glycosylation and co-receptor tropism

Since *env *length and glycosylation have been reported to correlate with mucosal transmission, including MTCT [15], we investigated these factors in our panel. In pairs M1001-P1024 and M1007-P1046, the median V1-V5 length of infant sequences was greater than maternal, while in pairs M1002-P1031, M1006-P1049, and M1003-P1189, the medians were similar (Table 3). The median number of V1-V5 PNGS was smaller in the infant sequences than in the mother's for pair M1002-P1031, greater for pair M1001-P1024, and equal in pairs M1007-P1046, M1006-P1049 and M1003-P1189 (Table 3). Statistical analysis did not indicate significant within-pair differences in the mean *env *length or glycosylation between maternal and infant clones. The V3 loop charge and glycosylation are predictive of co-receptor tropism [19,20]. Examination of charge and glycosylation of the V3 loops of our *env *clones did not reveal any CXCR4 (X4) tropic variants in our panel and only one mother (M1006), was predicted to harbor CCR5/CXCR4 dual tropic variants. Only CCR5 (R5) tropic maternal variants were transmitted to the infants (Table 3).

**Table 3:** Genotypic analyses of V1-V5 sequences

Subject*^a^*	V1-V5 length^b^*	V1-V5 PNGS^c^*	V3 charge	V3 glycan	V3 crown motif*^d^*	Tropism*^e^*
M1003	335	24 (23-25)	+3	Yes	APGR	R5
P1189	335	25 (25-25)	+3	Yes	APGR	R5
M1002	329 (329-333)	21 (20-24)	+3	Yes	GPGR	R5
P1031	329 (329-330)	19 (19-20)	+3	Yes	GPGR	R5
M1001	345 (342-347)	23 (22-25)	+2	Yes	GPGG, GPGR	R5
P1024	346 (345-346)	24 (23-24)	+2	Yes	GPGR	R5
M1007	328 (328-335)	23 (22-24)	+4	Yes	GPGR	R5
P1046	335	23 (21-23)	+4	Yes	GPGR	R5
M1006	332 (320-349)	24 (17-26)	+3 +4 +5	Yes, No	QPGR, QPGG	R5, R5/X4
P1049	332	24 (24-24)	+3	Yes	QPGR	R5

*^a^*M, Mother; P, Infant.

*^b^*Median length of the *env *V1-V5 region as median (min-max).

*^c^*Median number of potential N-linked glycosylation sites in the V1-V5 region as median (min-max).

*^d^*Dominant variant presented first.

*^e^*Tropism determined *in-vitro*. R5, CCR5; R5/X4, CCR5/CXCR4.

*p > 0.05. Pairwise differences between maternal and infant values were evaluated using Mixed Model ANOVA with mother-infant pairs included as random effects.

### Receptor and co-receptor requirements

**Figure 4:**
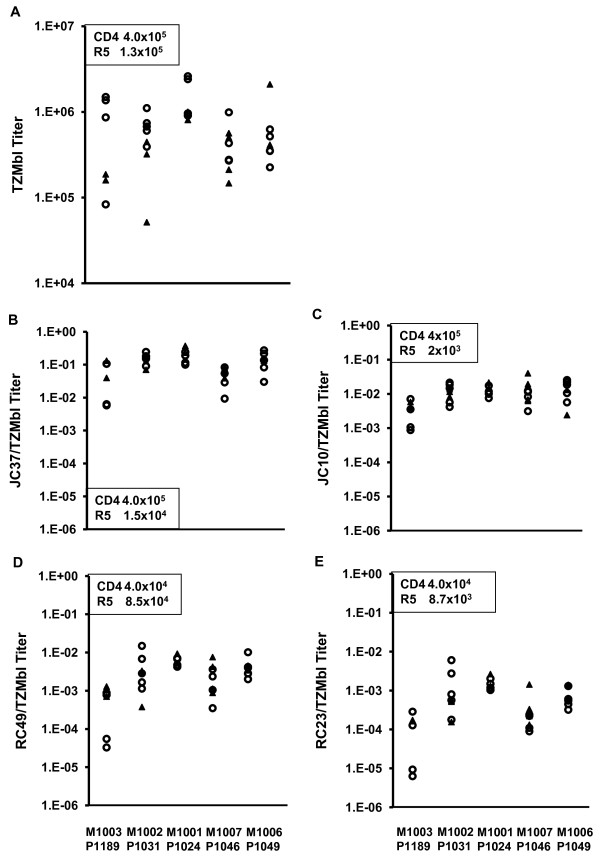
**Receptor and co-receptor requirements of cloned *env***. Pseudoviruses expressing cloned *env *were titered on HeLa cell lines engineered to express various levels of CD4 and CCR5. To normalize between different pseudovirus preparations, titers are expressed as a ratio of the titer on the cell line divided by the titer on TZMbl cells. **(A) **TZMbl, **(B) **JC37, **(C) **JC10, **(D) **RC49, **(E) **RC23. Results are an average of 3 independent experiments performed in duplicate. Average number of receptor and co-receptor molecules per cell as reported by Platt et al [21] is inset in the charts. Filled triangle = infant, empty circle = maternal. Pairwise statistical analysis performed using the Mixed Model ANOVA with mother-infant pairings included as random effects indicated that mean maternal and infant titers did not vary significantly across pairs.

The *in-silico *R5 tropism predictions were confirmed *in vitro *by comparing titers on the TZMbl and HIJ cell lines. TZMbl express both the CCR5 and CXCR4 co-receptors, while HIJ express CXCR4 but not CCR5 [21]. Pseudoviruses expressing the X4 tropic NL4.3 *env *and the R5 tropic SF162 *env *were used as controls; NL4.3 *env *infected both cell lines while SF162 *env *infected only TZMbl. All maternal and infant clones achieved high titers on TZMbl, but only one maternal clone (M1006 P1) infected both cell lines (Figure 4A).

The receptor (CD4) and co-receptor (CCR5) use of representative maternal and infant *env *clones (*n *= 35, Figure 1B) was then analyzed in depth. Pseudoviruses expressing these *env *were generated and titered on TZMbl cells (Figure 4A), and on additional HeLa cell lines expressing varying levels of CD4 and CCR5 [21] (Figure 4B-E). Infant viruses infected all cell lines tested. When pairwise comparisons were made, there was no significant difference between the mean infant and maternal titers on any cell line. All clones achieved highest titers on TZMbl cells, which express the highest levels of CD4 and CCR5. Titers decreased with decreasing levels of CD4 (Figure 4B verses D) or CCR5 (Figure 4B verses C, and D verses E), but were more sensitive to changes in CD4.

### Replication in primary macrophages and PBL

**Figure 5:**
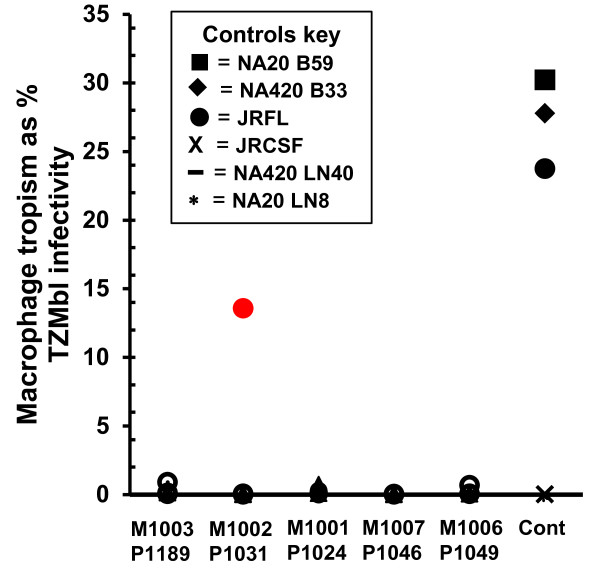
**Macrophage infectivity**. Pseudoviruses expressing cloned envelopes were titered on primary macrophage cultures. Macrophage infectivity is expressed as the percentage of the TZMbl titer achieved on macrophages. Data is representative of three independent assays performed in duplicate. Filled triangle = infant, empty circle = maternal. Clone M1002 G1 is highlighted red. Cont = Controls [42], see inset key: (macrophage tropic) NA20 B59, NA420 B33 and JRFL, (non-macrophage topic) JRCSF, NA420 LN40 and NA20 LN8.

We used two different approaches to evaluate the ability of maternal and infant viruses to replicate in primary macrophages. First, we investigated the ability of pseudoviruses expressing the *env *clones to mediate infection of primary macrophage cultures in a single round infection. All infant viruses exhibited low or no infectivity in monocyte derived macrophages (MDM); similarly, only a single maternal clone (M1002 G1) attained a high level of infection as compared to the non-macrophage tropic and highly macrophage tropic controls (Figure 5). Macrophage infectivity was further investigated by infecting matched donor MDM and PBLs with EGFP-tagged recombinant *env *clones from two randomly selected mother-infant pairs (Table 4). No fluorescence was detected in macrophage cultures throughout two weeks of infection while high levels of fluorescence were detected in each PBL infection. Measurement of HIV-1 p24 in the supernatants collected from cultures over the course of infection showed a steady decline from the input levels of p24 in macrophage infections, while PBL infections showed an increase. Altogether, these data demonstrate robust replication in PBL but uniformly poor replication in macrophages.

**Table 4:** Maternal and infant viruses replicate well in PBL but poorly in MDM

	Fluorescence		p24 ELISA*^a^*	
**Clone ID**	**MDM**	**PBL**	**MDM**	**PBL**

M1003 P16	No	Yes	No	Yes
M1003 D6	No	Yes	No	Yes
M1003 O1	No	Yes	No	Yes
M1003 Q4	No	Yes	No	Yes
P1189 F3	No	Yes	No	No
M-1007 Z8	No	Yes	No	Yes
M-1007 Q8	No	Yes	No	Yes
M-1007 Y7	No	Yes	No	Yes
P-1046 W2	No	Yes	No	Yes
P-1046 C4	No	Yes	No	Yes
P-1046 J1	No	Yes	No	Yes
P-1046 K1	No	Yes	No	Yes

*^a^*Increase in p24 antigen levels above input.

Infections with clones from the M1003-P1189 transmission pair were carried out at an MOI of 0.01, while those from the transmission pair M1007-P1046 were done at an MOI of 0.001.

### Sensitivity of envelope clones to neutralization by autologous maternal plasma

**Figure 6:**
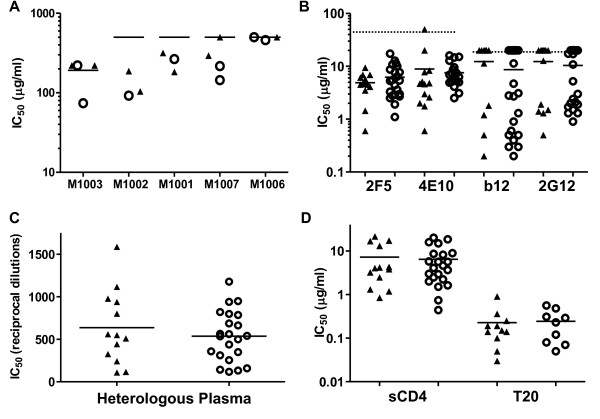
**Sensitivity of maternal and infant *env *to neutralization or inhibition**. The sensitivity of infant and maternal clones to **(A) **autologous maternal IgG, **(B) **NAbs, **(C) **pooled seropositive plasma and **(D) **entry inhibitors was determined using pseudovirus infection of TZMbl cells. **(A) **Neutralization IC_50 _of maternal and infant *env *clones. Lines indicate maximum concentration of IgG. **(B-D) **Values are an average of two different pseudovirus stocks run in the same experiment. Solid lines indicate infant and maternal means. Dotted lines in **(B) **indicate maximum concentration of NAb used. Filled triangle = infant, empty circle = maternal. Pairwise statistical analysis performed using the Mixed Model ANOVA with mother-infant pairings included as random effects indicated that mean maternal and infant IC_50 _did not vary significantly across pairs.

We assayed at least three clones from each mother-infant pair. Relatively high levels (≥ 100 μg/ml) of autologous maternal plasma IgG were required to neutralize maternal and infant viruses (Figure 6A). Statistical analysis did not indicate significant within-pair differences in the susceptibility of maternal and infant clones to neutralization by autologous maternal IgG.

### Sensitivity of envelope clones to neutralization by monoclonal antibodies and pooled seropositive plasma

Using a standardized assay [22,23], we tested the neutralization sensitivity profile of our pseudoviruses to a panel of well-established human NAbs, including b12 (CD4 binding site), 2G12 (carbohydrate-dependent) and the gp41 Membrane Proximal External Repeat (MPER) specific NAbs 2F5 and 4E10 (Figure 6B). No infant or maternal clone was resistant to 2F5. Only one clone was resistant to 4E10 (P1046 J1) and expressed the rare, resistance conferring, natural polymorphism F673L [24,25]. All clones from three infants were resistant to 20 μg of 2G12 and exhibited mutations eliminating one of five PNGS implicated in 2G12 binding [26]. In infant P1024, the mutation was N386D, in P1049 it was N392K, and in P1046 it was T292I. Most maternal clones from these pairs exhibited similar levels of 2G12 resistance, and displayed the corresponding mutations. Infants P1031, P1046 and P1049 had some clones resistant to 20 μg of b12, but each had one sensitive clone. A similar pattern of sensitive and resistant clones was seen in the corresponding mothers. When pairwise analyses were performed, we did not detect any trends for differential neutralization sensitivity between infant and maternal variants.

The neutralization sensitivity of the pseudoviruses to pooled heterologous plasma with high NAb activity was next determined (Figure 6C). Sensitivity varied over a 4-fold range within mother-infant pairs, but all infant and maternal viruses were sensitive to neutralization at plasma reciprocal dilutions ranging from 109 to 1588. Pairwise analysis failed to detect any trends for within-pairs differences in neutralization sensitivity to this reagent.

### Sensitivity of infant envelope clones to HIV-1 entry inhibitors

The sensitivity of infant clones to three HIV-1 entry inhibitors was evaluated (Figure 6 and data not shown). The inhibitors used were sCD4, T20 (fusion inhibitor) and Maraviroc (CCR5 antagonist). Since Maraviroc is a non-competitive inhibitor, we determined the MPI of our clones by this inhibitor. All clones were inhibited by >99% at concentrations exceeding 400 nM, indicating that none were resistant [27,28]. The NL4.3 *env *control exhibited a MPI of <2% (data not shown).

Infant clones were sensitive to T20 and sCD4, exhibiting IC_50 _ranges similar to the maternal. Mean T20 IC_50 _for infant clones was 0.23 μg/ml and 0.24 μg/ml for maternal. Soluble CD4 exhibited a mean IC_50 _of 7.24 μg/ml for infants and 6.43 μg/ml for mothers. No significant within-pair differences in sensitivity to these inhibitors were observed between maternal and infant viruses.

## Discussion

We generated full-length viable *env *clones from 5 mother-infant pairs and extensively characterized their V1-V5 genotypes and phylogeny. Phylogenetic analyses showed that infant sequences were more homogeneous than maternal viral sequences. The highest sequence diversity seen in the infants, 0.3%, fits well with the model of Keele *et al*. [9], which indicates that the maximum diversity expected within an individual shortly after infection with a single virus is 0.6%. Of the 8 transmitted/founder variants identified in the infants of our cohort, seven represented minor variants of the maternal quasispecies at the time of sampling, which was within a few weeks of transmission. These data support previous findings [13,14,29] suggesting a selective bottleneck during MTCT.

Consensus gp160 endpoint dilution sequences from two randomly selected infants were identical to those obtained by SGA. These results are compatible with a recent report that standard PCR and SGA provide similar measures of viral diversity when sufficient templates are analyzed [30].

Several groups have reported shorter hypervariable regions and fewer N-linked glycosylation sites in Clade C sexually (reviewed [31]) or maternally [32] transmitted viruses. Our data on Clade B viruses are compatible with others' work that did not find altered *env *length or glycosylation site number in transmitted Clade B viruses [33].

All infant clones were R5 tropic, consistent with numerous prior reports [34-36]. *Intrapartum *transmission of HIV-1 is hypothesized to occur across the mucosa, although the exact mechanisms have not been determined (reviewed [37,38]). Efficient HIV-1 infection usually requires the expression of relatively high levels of the CD4 receptor and CCR5 co-receptor on the surface of target cells [21,39]. However, levels of CD4 and CCR5 on mucosal and submucosal cell subsets can be much lower than on CD4^+ ^memory T cells [40]. Titration on cell lines expressing different levels of CD4 and CCR5 demonstrated efficient infection of cells with variable levels of these molecules. This finding is supported by our failure to observe any systematic differences in the sensitivity of maternal and infant *env *to inhibition by sCD4 or CCR5 inhibitors. It is also in agreement with a recent report that sexual HIV-1 transmission does not appear to select for viruses that can preferentially utilize lower levels of CD4 or CCR5 [39].

CCR5 co-receptor usage has traditionally been equated with macrophage-tropism. Peters *et al*. have recently clarified that not all R5 viruses are macrophage-tropic (reviewed [41]). Only 1 of 35 plasma-derived *env *clones achieved greater than 1% of their TZMbl titers on MDM. Our results are in agreement with prior data [42] demonstrating that peripheral blood viruses frequently exhibit low levels of macrophage infectivity, and that sexually transmitted R5 tropic variants replicate poorly in macrophages [43,44]. Finally, these findings support recent models of HIV-1 transmission, which suggest that cell subtypes other than macrophages are the first to encounter HIV-1 during mucosal transmission [44,45].

We screened our clones to determine their sensitivity to neutralization by a panel of well-characterized monoclonal Nabs. Sensitivity to these NAbs varied both between and within mother-infant pairs. Clones were uniformly sensitive to 2F5. Only one clone (from infant P1046) was resistant to 4E10; this clone exhibited the F673L natural polymorphism associated with resistance to this Nab [24]. All clones with 2G12 resistance correlated with loss of one of five PNGS that make up the 2G12 epitope.

At least two groups have reported that infant *env *clones are relatively resistant to neutralization by autologous maternal plasma [15,46]. Relatively high levels (IC_50 _≥ 100 μg/ml) of autologous maternal plasma IgG were required to neutralize maternal and infant viruses; however, all infant viruses were neutralized by pooled sera from HIV-1 infected individuals, implying that they were not inherently neutralization-resistant.

CCR5 antagonists are a potent new class of entry inhibitors. Since only R5 variants are vertically transmitted, CCR5 antagonists may be highly relevant to blocking MTCT; however their effectiveness against infant isolates has not been well characterized, and partial resistance to CCR5 antagonists in a treatment-naïve individual has been reported [28]. All *env *clones in our panel were sensitive to Maraviroc. All infant clones were also sensitive to T20 and sCD4, and no significant differences in sensitivity were seen between maternal and infant viruses. The latter is in contrast with data from Keele *et al*. [9], who demonstrated significantly higher IC_50 _values for T1249, a fusion inhibitor with a mechanism of action similar to T20, among viruses from acutely infected as compared to chronically infected subjects.

## Conclusions

Although we have a relatively small sized patient cohort, the results of our extensive genotypic and phenotypic studies confirm that clade B MTCT occurs across a selective bottleneck, and that neither *env *length nor glycosylation appear to play a role in this selection. Utilization of low receptor and co-receptor levels for entry likewise does not appear to play a major role in the selective bottleneck during vertical transmission of HIV-1 clade B. Most intriguingly, R5 tropic maternal and infant *env *exhibited poor macrophage infectivity. Relatively high levels (IC_50 _≥ 100 μg/ml) of autologous maternal plasma IgG were required to neutralize maternal and infant viruses. Maternal and infant clones were equally sensitive to pooled heterologous plasma, implying that inherent neutralization resistance is unlikely to be a major factor controlling the selective bottleneck. Infant clones were variably sensitive to neutralization by monoclonal antibodies but uniformly sensitive to HIV-1 entry inhibitors. Together, our findings provide further insight into the selective pressures influencing the genetic bottleneck during vertical transmission of HIV-1 and may help inform the future development of therapies to prevent MTCT.

## Materials and methods

### Study population

Plasma samples were obtained from 5 HIV-1 clade B infected women and their infants (Table 1). Maternal samples were obtained at or within a month of delivery. None of the mothers exhibited opportunistic infections or AIDS-defining illnesses. All five infants were infected at delivery, based on standard definitions [5]. Most *intrapartum *transmission is thought to occur across the mucosa although the exact mechanisms have not been determined (reviewed [37,38]). None of the infants were breastfed. Infant samples were obtained within 2 months of delivery and represent the first time point at which HIV-1 was detected in the infants by viral isolation or the detection of nucleic acids.

### PCR amplification and generation of functional envelope (gp160) clones via endpoint dilution

Viral RNA was extracted from 50-200 μl of plasma using the Roche High Pure Viral RNA Kit (Roche Pharmaceuticals, Basel, Switzerland). Eluted RNA was treated with 1 μl of RNasin Plus RNase inhibitor (Promega Biosciences, San Luis Obispo, CA), then aliquoted and stored at -80°C. Full-length HIV-1 gp160 was amplified directly from the viral RNA by endpoint dilution nested RT-PCR. To identify the endpoint dilutions, RT-PCR was performed in octuplet on two fold serial dilutions of each viral RNA extract until a dilution was reached where not more than three of eight wells showed product. Outer and inner primer pairs were the same as reported by Wei *et al*. [47]. RT-PCR was performed using the Superscript One Step RT-PCR for Long Templates kit (Invitrogen Life Technologies, Carlsbad, CA). Conditions for the outer PCR were as follows: 45°C for 30 min, 94°C for 2 min, 40 cycles of 94°C for 15 sec, 52°C for 30 sec, 68°C for 3 min, with a final extension at 72°C for 10 min. Inner PCR was performed using the Platinum Taq DNA Polymerase HighFidelity kit (Invitrogen Life Technologies, Carlsbad, CA). Conditions for the inner PCR were as follows: 94°C for 2 min, 40 cycles of 94°C for 15 sec, 55°C for 30 sec, 68°C for 3 min, with a final extension at 72°C for 10 min. The ~3 kb *env *amplicons were sub-cloned into the pcDNA3.1/V5-His TOPO TA vector (Invitrogen Life Technologies, Carlsbad, CA) using the manufacturer's instructions. Colonies containing full length inserts in the correct orientation were identified by a PCR screen; their functionality was determined using syncytia as a readout by the addition of HeLa cells expressing CD4 and CCR5 (TZMbl a.k.a. JC53BL [21,48]) to monolayers of 293T cells [49] transfected with the molecularly cloned *env *[50]. At least 10 functional *env *clones were obtained from each subject, with each clone originating from an independent endpoint dilution PCR.

### Single genome amplification (SGA)

SGA was performed as described by Salazar-Gonzalez *et al*. [10]. Briefly, viral RNA extracted as above was reverse transcribed to single-stranded cDNA using primer OFM1. The cDNA was diluted in 96 well plates such that less than 30% of the reactions yielded amplified product. Nested PCR was then carried out using primers Vif1 and OFM19 for the outer step, and EnvA and EnvN for the inner. All correctly sized products were purified and sequenced.

### DNA sequencing, phylogenetic analysis and clone selection

The V1-V5 regions of all viable molecular *env *clones were sequenced using BigDye Terminator chemistry. Sequences were assembled using the Vector NTI software (Invitrogen Life Technologies, Carlsbad, CA). *Env *sequences from each subject were aligned using ClustalW in the software package BioEdit http://www.mbio.ncsu.edu/BioEdit/BioEdit.html, and trees were constructed using the neighbor joining method [51] implemented in Mega http://www.megasoftware.net using Kimura's correction [52] and 1000 iterations of Bootstrap analysis, and the maximum likelihood method with 500 iterations of Bootstrap analysis implemented in PhyML http://www.hiv.lanl.gov. Phylogeny was confirmed using the Highlighter software program http://www.hiv.lanl.gov. Potential N-Linked glycosylation sites (PNGS) were identified using the N-Glycosite program http://www.hiv.lanl.gov. The V3 loop charge was determined by comparing the number of positively charged (Aspartic Acid and Glutamic Acid) to negatively charged (Lysine and Arginine) amino acid residues.

### Pseudovirus production and titration

Pseudoviruses were made by co-transfecting exponentially dividing 293T cells with a 1:2 ratio of *env *and pSG3Δ*env *backbone (NIH AIDS Research and Reference Reagent Program [47,53]) using Polyethylenimine (Polysciences, Warrington, PA) as the transfection reagent. Pseudoviral titers were determined using single round infection of TZMbl cells essentially as described [23] except that β-galactosidase staining rather than luminescence was used as the readout. Cells developed using the β-galactosidase readout were counted on an automated ELISPOT reader (See Additional File 1; Supplemental Methods). The titers were expressed as spot forming units per ml (sfu/ml). Assays utilizing luminescence gave results very similar to those determined by using β-galactosidase (data not shown). Titrations were performed at least twice for each pseudovirus.

### Construction of replication competent fluorescently tagged HIV-1

A fluorescently tagged, replication competent HIV-1 backbone was obtained from Dr. Matthias Dittmar (Centre for Infectious Disease, Institute of Cell and Molecular Science, Barts and The London School of Medicine and Dentistry). Plasmids encoding selected infant and maternal *env *in this backbone were generated as described [54]. Briefly, we used the plasmid TN6GΔ, which encodes the full length NL4.3 HIV-1 clone with the *nef *gene replaced by EGFP and has unique restriction sites (*Bst*EII and *Nco*I) in the *env *gene available for inserting heterologous *env*. The complementary restriction sites were introduced into selected infant and maternal *env *clones and used for directional sub-cloning into TN6GΔ. Live, fluorescently tagged virus was produced using essentially the same protocol as for the pseudovirus described above.

### Cell line, macrophage, and peripheral blood lymphocyte (PBL) titrations and infections

Receptor and co-receptor requirements of pseudoviruses were determined by titration on HeLa cells engineered to express various levels of the CD4 receptor and CCR5 and CXCR4 co-receptors [21]. *In silico *predicted CCR5 tropism was confirmed by titration on the HIJ HeLa cell line, which expresses CD4 and CXCR4 but no CCR5 [21], using pseudoviruses expressing the CXCR4 tropic NL4.3 *env *[55] and the CCR5 tropic SF162 *env *(NIH AIDS Research and Reference Reagent Program Catalog # 10463) as controls. Titrations were performed as described [50] utilizing anti-p24 immunostaining as the infectivity readout. To determine macrophage infectivity, elutriated monocytes were re-suspended in medium containing macrophage colony stimulating factor and cultured for seven days before use for pseudoviral titrations essentially as described [50]. Each pseudovirus was tested in duplicate in 3 independent assays. To normalize between different pseudoviral preparations, all titers are expressed as a ratio of the titer on the cell line or macrophage culture divided by the titer on TZMbl cells.

For infection of PBLs, fresh PBLs were maintained in RPMI 1640 medium supplemented with 10% FBS, stimulated with phytohemagglutinin (5 μg/ml) for 2 days, and interleukin-2 (10 U/ml) for a further 2 days prior to infection. PBLs were infected with live virus at an MOI of 0.01 or 0.001 as indicated (based on TZMbl or PBL titers as appropriate) and carried for seven days before being read for HIV-1 positive cells by anti-p24 immunostaining or flow cytometry.

### Neutralization and inhibition assays

Neutralization and inhibition assays using human monoclonal antibodies, pooled HIV-positive patient sera, autologous maternal plasma, or HIV-1 entry inhibitors were performed as previously described [22,23,56-58], using 200 sfu of pseudovirus to infect TZMbl cells, with residual infection measured using the β-galactosidase readout. To determine the activity of CCR5 antagonists, the assay was modified such that cell monolayers were incubated with serial dilutions of the inhibitors for one hour before the addition of virus. Following the addition of virus, plates were incubated overnight and the media was replaced with fresh un-supplemented media. Pseudoviral stocks expressing well-characterized *env *from the NIH AIDS Research and Reference Reagent Program Standard Reference Panels of Subtype B or C HIV-1 *Env *Clones [22,56,57] were used in every experiment and showed low intra-assay variation, and values similar to those reported [56,57] (data not shown). Monoclonal NAbs b12, 2G12, 4E5, and 2F5 were obtained from the NIH AIDS Research and Reference Reagent Program; an additional aliquot of b12 was generously provided by Dr. Dennis Burton (Scripps Research Institute). The maximum b12 and 2G12 antibody concentration used in neutralization assays was 20 μg/ml, while 4E10 and 2F5 were used at 50 μg/ml. HIV-1 entry inhibitors soluble CD4 (sCD4, Progenics Pharmaceuticals, Tarrytown, NY), Enfuvirtide (T20, Roche, Palo Alto, CA) and Maraviroc (obtained through the NIH AIDS Research and Reference Reagent Program) were also evaluated in these assays. As Maraviroc is a non-competitive inhibitor, resistance was determined by changes in the maximal percent inhibition (MPI) [59]. The MPI of our clones was determined by observing plateaus in the inhibition curves [59] at saturating concentrations of Maraviroc (up to 4000 nM) [28,59]. All plasma was heat inactivated at 56°C for 30 minutes before use. Sero-negative plasma was used as a negative control and showed no neutralization activity at 1:15 dilution. Pseudovirus expressing murine leukemia virus (MLV) *env *was used as a non-specific neutralization control and generally failed to be inhibited by the highest concentration of plasma used. Since we have previously observed non-specific neutralization of the MLV control and available plasma volumes were limited, we used purified Immunoglobulin G (IgG) in our autologous neutralization assays (see below). For all antibodies and inhibitors, the 50% inhibitory concentration (IC_50_) relative to untreated control infections was determined from plots generated using the sigmoidal fit function of the OriginPro 7.5 SRO v7 software package [60] (See Additional File 1; Supplemental Methods).

### Autologous neutralization using purified IgG

IgG was purified from plasmas using the Nab Protein Spin Kit (Thermo Scientific, Rockford, IL) according to the manufacturer's protocol. Elution fractions one and two were pooled and dialyzed in culture media using the Slide-A-Lyzer Dialysis Kit, 10 K MWCO (Pierce Biotechnology, Rockford, IL). The amount of IgG in the dialyzed extracts and the original maternal plasma was quantified using the Human IgG ELISA Kit (ZeptoMetrix Corporation, Baffalo, NY). Autologous neutralizations were set up at an initial IgG concentration of 0.5 mg/ml.

### Statistical analyses

Pairwise differences between maternal and infant values were evaluated using Mixed Model ANOVA [61] with mother-infant pairs included as random effects. The analyses were performed using the Proc Mixed procedure [62] in the SAS statistical Software package (SAS Inc, NC, USA). Significance was reported when p ≤ 0.05.

### Nucleotide sequence accession numbers

Nucleotide sequence accession numbers are available under [GenBank: HM368224 - HM368258].

### Ethics Statement

Ethical approvals were obtained from the University of Massachusetts Medical School, Institutional Review Board for Human Subjects. Informed, written consent was obtained from each of the women for their own and their infants' participation prior to enrollment in this study.

## Competing interests

The authors declare that they have no competing interests.

## Authors' contributions

MK, FB, MS, PC, KL conceived and designed experiments. MK and FB performed the experiments. MK, FB, MS, PC and KL analyzed the data. PC, KL, and JLS contributed samples and reagents. MK, MS and KL wrote the manuscript. All authors read and approved the manuscript.

## Supplementary Material

Additional file 1 **Supplemental Methods**. Detailed methodology for the PCR screen, β-galactosidase readout and 50% inhibitory concentration calculations. Click here for file
