# Supplementary material for: Genotypic and functional properties of early infant HIV-1 envelopes
Source: Retrovirology. 2011 Aug 15;8:67. doi: 10.1186/1742-4690-8-67 (PMC3189118; doi:10.1186/1742-4690-8-67)
Supplement: Additional file 1 — Supplemental Methods. Detailed methodology for the PCR screen, β-galactosidase readout and 50% inhibitory concentration calculations. [file 1742-4690-8-67-S1.PDF]

Supplemental methods

PCR screen. Colonies containing full length inserts in the correct orientation were identified by a PCR screen using GoTaq Green Master Mix (Promega Biosciences, San Luis Obispo, CA) and the 5' primer pair T7 and *env*-572 (TAG GCC AGT AGT ATC AAC TCA ACT) and the 3' pair BGH and *env*-587 (AAT CTC CTA CAG TAT TGG AGT CAG). Conditions for both reactions were as follows: 95°C for 5 min, 25 cycles of 95°C for 1 min, 50°C for 1min, 72°C for 1 min, with a final extension at 72°C for 5 min.

β-galactosidase readout. As an alternative to using luminescence for the TZMbl neutralization assay readout, we utilized ß-galactosidase activity. Following the final 24-hour incubation step, the plates were fixed with ice-cold gluteraldehyde, washed with PBS and developed with X-gal in yellow PBS. Blue stained cells and cell-clusters were enumerated mechanically on an ELISPOT reader using the Immunospot 4.0.16 software (Cellular Technologies, Ltd., Cleveland, OH). Using this method allowed us to directly visualize and count foci forming units. This method leaves TZMbl monolayers intact making trouble shooting easier, and allows long-term storage and re-reading of plates. X-gal and yellow PBS are also significantly less expensive than luminometer substrates. In several side-by-side assays we found the differences in the results between the two readouts were of the same magnitude as the variation between assays using the same readout out (data not shown).

50% inhibitory concentration calculations. Background foci counts from empty wells were subtracted from both the averaged counts of triplicate antibody

dilution wells and the averaged counts from octuplet cell-only and uninhibited-virus control wells. Percentage inhibition values were then calculated using the formula:

$$\left(1-\left[\frac{(AntibodyDilution)-(CellOnly)}{(UninhibitedVirus)-(CellOnly)}\right]\right)X100$$

Graphs were prepared by plotting $\log_{10}$ Antibody Concentration vs. Percentage Inhibition for each antibody/virus combination using the sigmoid fit function of the OriginPro 7.5 SRO v7 software package (www.originlab.com). EC_{50} values calculated by the OriginPro software were used as final IC_{50} determinations.
